# Zoonotic Neglected Tropical Diseases: New Approaches to Combat Old Enemies

**DOI:** 10.1155/2014/914248

**Published:** 2014-06-05

**Authors:** Fabio Ribeiro Braga, Pedro Mendoza de Gives, Adolfo Paz Silva, Filippe Elias de Freitas Soares, Jackson Victor de Araújo

**Affiliations:** ^1^Department of Veterinary, Federal University of Viçosa, University Campus s/n, P.O. Box 3657000, Viçosa, MG, Brazil; ^2^Department of Helminthology, CENID-Veterinary Parasitology, INIFAP Federal Highway Cuernavaca, Cuautla No. 8534, P.O. Box 62550, Jiutepec, MO, Mexico; ^3^Veterinary Faculty, University of Santiago de Compostela, 27002 Lugo, Spain

Neglected tropical diseases are a group of tropical infections which are especially endemic in low-income populations in developing regions of Africa, Asia, and the Americas. Different groups define the set of diseases. In this context, we mention roundworms (ascariasis), whipworms (trichuriasis), hookworms (necatoriasis and ancylostomiasis), trematodes (schistosomiasis), lymphatic filariosis, leishmaniasis, American trypanosomiasis, hanseniasis, African trypanosomiasis, Guinea worm (dracunculiasis), and Buruli ulcer. Due to the fact that thousands of people become affected, the social and economic impacts of these diseases are tremendous. Together, they cause an estimated 500,000 to 1 million deaths per year and cause an overall charge higher than the HIV-AIDS, possibly due to the lack of adequate tools for their diagnosis and treatment. They affect the poorest populations in the least developed countries of the world and therefore do not constitute a lucrative market for the pharmaceutical industries.

It should also be emphasized that these diseases represent an enemy that takes advantage of social and economic fragility. However, articles which also cover other diseases such as malaria, toxoplasmosis, and cryptosporidiosis were welcomed. In another context, in this special section, we call attention to research that focuses on the Trematoda* Paragonimus westermani* (paragonimiasis).

In this special edition some proposals for advances in controlling and reducing the risk of these diseases have been reported, with an approach to biotechnology and biomedicine. Moreover, the main objective was to bring to the “light of knowledge” further research to combat neglected tropical diseases.

Thus, the quick growth in the adoption of the concept of a healthcare, in particular in developed countries over the last six years, was demonstrated, where the interesting focus of this research was to observe the advantages and benefits to the best approach to combating zoonoses [1]. In another moment, the edition included an experience in the field of campylobacteriosis in chickens and, recording with that, the concern with poultry production, which in many situations may be associated with other creations of domestic animals and, with that, the close association and opportunity for movement of* Campylobacter* spp. among these host species (“*MLST genotypes and antibiotic resistance of Campylobacter spp. isolated from poultry in Grenada*”).

In a study conducted in Brazil, valuable information in the field of diseases and pathogens of buffalos in the northern region has been reported and may benefit management programs and control of disease in these animals [2].

In the field of the study of protozoa, two investigations have been reported, one evaluating the direct effect of two new compounds on the viability and infectivity of tachyzoites of* Toxoplasma* [3] and the second about the cellular immunity, especially for the remission of lesions and protection against infection from cutaneous leishmaniasis [4]. Finally, in the area of combating helminths by natural antagonists stood out the research of filamentous fungi from the genera ovicides and predators to combat liver* Fasciola hepatica,* causing fasciolosis, an important emerging zoonosis (“*Mixed production of filamentous fungal spores for preventing soil-transmitted helminth zoonoses: a preliminary analysis*”).

*Fabio Ribeiro Braga* *Pedro Mendoza de Gives* *Adolfo Paz Silva* *Filippe Elias de Freitas Soares* *Jackson Victor de Araújo*
